# Comparison of suspension MDCK cells, adherent MDCK cells, and LLC‐MK2 cells for selective isolation of influenza viruses to be used as vaccine seeds

**DOI:** 10.1111/irv.12694

**Published:** 2019-10-25

**Authors:** Yuichi Harada, Hitoshi Takahashi, Heidi Trusheim, Bernhard Roth, Katsumi Mizuta, Asumi Hirata‐Saito, Teruko Ogane, Takato Odagiri, Masato Tashiro, Norio Yamamoto

**Affiliations:** ^1^ Influenza Virus Research Center National Institute of Infectious Diseases Tokyo Japan; ^2^ Novartis Vaccines and Diagnostics GmbH Marburg Germany; ^3^ Yamagata Prefectural Institute of Public Health Yamagata Japan; ^4^ Tochigi Prefectural Institute of Public Health and Environmental Science Utsunomiya Japan; ^5^ Department of Infection Control Science Graduate School of Medicine Juntendo University Tokyo Japan; ^6^ Present address: IDT Biologika GmbH Dessau‐Roßlau Germany; ^7^ Present address: GSK Vaccines GmbH Marburg Germany

**Keywords:** adventitious virus, cell‐based vaccine, influenza, Madin‐Darby canine kidney cell line, vaccine seed virus

## Abstract

**Background:**

Cell‐based influenza vaccines can solve the problem of the frequent occurrence of egg adaptation–associated antigenic changes observed in egg‐based vaccines. Seed viruses for cell‐based vaccines can be prepared from clinical specimens by cell culture; however, clinical samples risk harboring respiratory viruses other than influenza virus. Therefore, it is necessary to investigate the patterns of co‐infection in clinical samples and explore whether cell culture technology can selectively propagate influenza viruses from samples containing other respiratory viruses.

**Methods:**

A total of 341 clinical specimens were collected from patients with influenza or influenza‐like illness and analyzed by ResPlex II assay to detect 18 respiratory viruses. The patterns of co‐infection were statistically analyzed with Fisher's exact test. The samples with double or triple infections were passaged in suspension MDCK cells (MDCK‐S), adherent MDCK cells (MDCK‐A), and LLC‐MK2D cells. Cell‐passaged samples were analyzed by ResPlex II assay again to investigate whether each cell line could amplify influenza viruses and eliminate other respiratory viruses.

**Results:**

Double infections were detected in 8.5% and triple infections in 0.9% of the collected clinical specimens. We identified four pairs of viruses with significant correlation. For all samples with double and triple infection, MDCK‐S and MDCK‐A could selectively propagate influenza viruses, while eliminating all contaminating viruses. In contrast, LLC‐MK2D showed lower isolation efficiency for influenza virus and higher isolation efficiency for coxsackievirus/echovirus than MDCK‐S and MDCK‐A.

**Conclusions:**

Both MDCK‐S and MDCK‐A are considered suitable for the preparation of influenza vaccine seed viruses without adventitious agents or egg‐adaptation mutations.

## 1 INTRODUCTION

Influenza virus is highly transmissible and causes mild to severe illness, including high fever, headache, myalgia, and pneumonia. Annual influenza epidemics worldwide cause approximately three to five million cases of severe illness and 290 000‐650 000 deaths every year, resulting in a great social impact.^1^ The economic burden of influenza has been estimated to be $47.2‐$149.5 billion per year in the United States.^2^, ^3^

Influenza vaccine, one of the most effective measures to prevent influenza virus infection, is mainly produced in embryonated chicken eggs. However, influenza viruses propagated in eggs frequently acquire antigenic alteration through host adaptation,^4^, ^5^, ^6^, ^7^, ^8^, ^9^ and this change would pose a serious challenge owing to the reduction in vaccine effectiveness.

Cell‐based vaccines can potentially solve the problem of antigenic changes associated with egg adaptation. Vaccine seed viruses, for cell‐based vaccines, can be prepared from clinical specimens with cell cultures; however, clinical samples have a risk of harboring respiratory viruses other than influenza viruses. Moreover, mammalian cells might amplify contaminating human viruses more easily than embryonated chicken eggs owing to host similarity. Therefore, it is necessary to investigate the patterns of co‐infection present in clinical samples and explore whether cell culture technology can selectively propagate influenza viruses from samples containing other respiratory viruses.

For the propagation of influenza viruses, cell lines such as Madin‐Darby canine kidney (MDCK) cells, Vero cells, LLC‐MK2 cells, and PER.C6 cells have been commonly used.

The MDCK cell line was established from the kidney of a healthy cocker spaniel dog in 1958 and has a long history in the studies of influenza viruses. The conventional MDCK cell with adherent growth (MDCK‐A) is a good candidate for the preparation of vaccine seed viruses, since it supports efficient growth of human influenza viruses.^10^, ^11^

The MDCK33016PF suspension cell line, designated as MDCK‐S in this paper, was first developed and utilized to produce seasonal influenza vaccines.^11^, ^12^, ^13^ Suspension cells are superior to adherent cells owing to the following advantages: simpler culture process without micro‐carrier beads, lower cost, and higher virus yield. Therefore, MDCK‐S could be a suitable substrate for influenza vaccine seed preparation.

LLC‐MK2, established from the kidney of a healthy rhesus monkey, has also been used to propagate a variety of viruses, including the influenza virus.^14^, ^15^, ^16^ LLC‐MK2D, which is a sub‐line of LLC‐MK2, was proven to be non‐tumorigenic in nude mice and free of specific adventitious agents. This cell line could be a promising candidate for the preparation of vaccine seed viruses, since its safety is confirmed and it can be used in practical applications with little delay.

In this study, we analyzed the pattern of co‐infection of respiratory viruses in clinical specimens and evaluated the ability of MDCK‐S, MDCK‐A, and LLC‐MK2D cells to propagate influenza viruses while eliminating other respiratory viruses.

## 2 MATERIALS AND METHODS

### 2.1 Clinical specimens

We selected 341 clinical specimens from the patients diagnosed as having influenza or influenza‐like illness based on their clinical symptoms and/or the results of rapid tests for influenza. These patients visited the hospitals during the 2006/2007 influenza season (from January 2007 to May 2007), 2007/2008 influenza season (from December 2007 to April 2008), or 2008/2009 influenza season (from September 2008 to February 2009) in Japan. Nasal or pharyngeal samples were collected using UTM 360C kits (Copan Italia, Brescia, Italy) or transport medium consisting of minimum essential medium (MEM) supplemented with 0.5% gelatin, 100 units/mL of penicillin, and 100 µg/mL of streptomycin. These specimens were aliquoted and stored at −80°C until use. The study protocol was approved by the ethics committee of the National Institute of Infectious Diseases, Japan.

### 2.2 Cell culture and virus passaging

MDCK‐S cells were cultured in 500‐mL disposable spinner flasks (Corning, Corning, NY, USA) with 100 mL of chemically defined medium (CDM) at 37°C, 5% CO_2_, and 100 rpm on a shaking platform (MIR‐S100C; Sanyo, Osaka, Japan). MDCK‐A and LLC‐MK2D cells were subcultured in MEM supplemented with 10% fetal bovine serum (FBS) in a 75‐cm^2^ culture flask at 37°C under 5% CO_2_.

All adventitious virus‐positive specimens were inoculated into the cultures of these cells. For virus isolation using MDCK‐S, the infection medium was prepared with 37.5 µg/mL of neomycin (Gibco, Carlsbad, CA, USA) to prevent bacterial contamination from specimens and 1 µg/mL of TrypZean (Sigma‐Aldrich, St. Louis, MO, USA) to support viral growth. The density of MDCK‐S in the infection medium was adjusted to 1 × 10^6^ cells/mL, and 5 mL of cell suspension was distributed to 50‐mL filter‐capped tubes. For virus isolation with MDCK‐A and LLC‐MK2D, 1.5 × 10^6^ cells of MDCK‐A and 3 × 10^5^ cells of LLC‐MK2D were seeded in dishes of 60‐mm diameter 3 days before inoculation and maintained in MEM with 10% FBS until further use. OptiPRO serum‐free medium containing 4 mM l‐Glu, 37.5 µg/mL neomycin, and 1 µg/mL TrypZean (for MDCK‐A) or 5 µg/mL Trypsin Acetylated (for LLC‐MK2D) was used to prepare the virus isolation medium. Fifty microliters of clinical specimens was inoculated into the cultures of the three cell lines and incubated under 5% CO_2_ at 34°C for 72 hour. Supernatants were harvested and passaged further. After two passages, ResPlex II assays were performed again to investigate the presence or absence of influenza and other respiratory viruses.

### 2.3 ResPlex II assay

Viral nucleic acids in each sample were extracted using the QIAamp MinElute Virus Spin Kit (QIAGEN GmbH, Hilden, Germany) or QIAamp viral RNA mini kit (QIAGEN). The ResPlex II v2.0 kit (QIAGEN) was used, according to the manufacturer's instructions, to detect 18 human respiratory viruses as follows: respiratory syncytial virus type A (RSVA), respiratory syncytial virus type B (RSVB), influenza A virus (INFA), influenza B virus (INFB), parainfluenza virus type 1, parainfluenza virus type 2, parainfluenza virus type 3, parainfluenza virus type 4, human metapneumoviruses A and B, coxsackievirus/echovirus (CVEV), rhinovirus (RHV), adenovirus type B (ADVB), adenovirus type E, coronavirus NL63 (NL63), coronavirus HKU1, coronavirus 229E (229E), coronavirus OC43 (OC43), and bocavirus (BocV). The principle of this assay is based on multiplex RT‐PCR in combination with fluorescence detection of specific PCR amplicons on the LiquiChip 200 Workstation using QIAplex MDD software.^13^

## 3 RESULTS

A total of 341 samples from patients diagnosed with influenza or influenza‐like illness were analyzed for 18 respiratory viruses using the ResPlex II assay. The number of samples positive for INFA, INFB, and other respiratory viruses was 227 (66.6%), 85 (24.9%), and 38 (11.1%), respectively. Double infections were detected in 29 samples (8.5%) and triple infections in 3 samples (0.9%) (Table 1). The combination of viruses most frequently identified was INFA and INFB in the double infection group (8/29 double‐positive samples) and INFA, RHV, and CVEV in the triple infection group (2/3 triple‐positive samples).

**Table 1:** Distribution of detected viruses

Detected virus	Number of virus‐positive samples	Rate of virus‐positive samples (%)
One virus alone	283	82.99
INFA	200	58.65
INFB	75	21.99
CVEV	3	0.88
OC43	2	0.59
RSVA	2	0.59
NL63	1	0.29
Two viruses	29	8.50
INFA, INFB	8	2.35
INFA, OC43	6	1.76
INFA, CVEV	4	1.17
INFA, RHV	3	0.88
INFA, NL63	2	0.59
INFA, BocV	1	0.29
INFB, 229E	1	0.29
INFB, ADVB	1	0.29
RHV, CVEV	2	0.59
RSVB, CVEV	1	0.29
Three viruses	3	0.88
INFA, RHV, CVEV	2	0.59
INFA, OC43, ADVB	1	0.29
Any virus	324	95.01
No virus	17	4.99
Total	341	100.00

To examine if there were specific patterns of correlation between the detected viruses, all virus pairs in Table 1 were evaluated with Fisher's exact test for 2 × 2 tables. In addition, the detection rate of one virus in the background of another virus‐positive sample was compared with that in the background of all samples, to determine whether correlation was positive or negative. We identified four pairs of viruses with significant association (*P* < .05), where INFA‐INFB and INFB‐CVEV showed negative correlation and CVEV‐RHV and CVEV‐RSVB exhibited positive correlation (Table 2). However, influenza viruses were not significantly correlated with most of the other respiratory viruses. These results suggest that INFA and INFB would mutually interfere in each other's infection, whereas infection with influenza and other viruses would occur independently in most cases.

**Table 2:** Virus pairs with a statistically significant correlation*

Virus 1	Virus 2	Detection rate of virus 1 (%)		Detection rate of virus 2 (%)		*P* value	Interpretation
		Background: all samples	Background: virus 2‐positive samples	Background: all samples	Background: virus 1‐positive samples		
INFA	INFB	70.06	9.41	26.23	3.52	2.20E‐16	Negative correlation
INFB	CVEV	26.23	0.00	3.70	0.00	4.09E‐02	Negative correlation
CVEV	RHV	3.70	57.14	2.16	33.33	3.85E‐07	Positive correlation
CVEV	RSVB	3.70	100.00	0.31	8.33	3.70E‐02	Positive correlation

* *P* < .05.

To investigate whether MDCK‐S, MDCK‐A, and LLC‐MK2D can selectively amplify influenza virus in the presence of other respiratory viruses, co‐infection samples containing influenza virus plus other viruses were inoculated and passaged in the three cell lines. From all samples with triple or double infection, MDCK‐S and MDCK‐A could selectively allow proliferation of influenza viruses while eliminating all contaminating viruses (Table 3). Particularly, the isolation rates of influenza viruses were significantly higher than those of co‐existing viruses in the groups of double infection 1 (INFA and OC43, *P* < .01), double infection 2 (INFA and CVEV, *P* < .01), and double infection 3 (INFA and RHV, *P* < .05). For LLC‐MK2D, influenza viruses were isolated from the groups of double infection 1 and double infection 3 without propagation of adventitious viruses; however, the differences in detection rates between influenza virus and others were not significant. In the double infection 2 group, an isolate of CVEV was obtained from four specimens (25%), although no influenza virus was amplified during cell passages.

**Table 3:** Cell passages of field samples positive for influenza virus plus other viruses

Group	Target viruses	Clinical samples (positive/total)	MDCK‐S‐passaged samples (positive/total)	MDCK‐A‐passaged samples (positive/total)	LLC‐MK2D‐passaged samples (positive/total)
Triple infection 1	INFA	100% (2/2)	100% (2/2)	100% (2/2)	0% (0/2)
	RHV	100% (2/2)	0% (0/2)	0% (0/2)	0% (0/2)
	CVEV	100% (2/2)	0% (0/2)	0% (0/2)	0% (0/2)
Triple infection 2	INFA	100% (1/1)	100% (1/1)	100% (1/1)	0% (0/1)
	OC43	100% (1/1)	0% (0/1)	0% (0/1)	0% (0/1)
	ADVB	100% (1/1)	0% (0/1)	0% (0/1)	0% (0/1)
Double infection 1	INFA	100% (6/6)	100% (6/6)*	100% (6/6)*	16.7% (1/6)
	OC43	100% (6/6)	0% (0/6)	0% (0/6)	0% (0/6)
Double infection 2	INFA	100% (4/4)	100% (4/4)*	100% (4/4)*	0% (0/4)
	CVEV	100% (4/4)	0% (0/4)	0% (0/4)	25% (1/4)
Double infection 3	INFA	100% (3/3)	100% (3/3)**	100% (3/3)**	33.3% (1/3)
	RHV	100% (3/3)	0% (0/3)	0% (0/3)	0% (0/3)
Double infection 4	INFA	100% (2/2)	100% (2/2)	100% (2/2)	0% (0/2)
	NL63	100% (2/2)	0% (0/2)	0% (0/2)	0% (0/2)
Double infection 5	INFA	100% (1/1)	100% (1/1)	100% (1/1)	0% (0/1)
	BocV	100% (1/1)	0% (0/1)	0% (0/1)	0% (0/1)
Double infection 6	INFB	100% (1/1)	100% (1/1)	100% (1/1)	0% (0/1)
	229E	100% (1/1)	0% (0/1)	0% (0/1)	0% (0/1)
Double infection 7	INFB	100% (1/1)	100% (1/1)	100% (1/1)	0% (0/1)
	ADVB	100% (1/1)	0% (0/1)	0% (0/1)	0% (0/1)

* The detection rate of influenza virus was significantly higher than that of the other co‐infected viruses (*P* < .01).

** The detection rate of influenza virus was significantly higher than that of the other co‐infected viruses (*P* < .05).

To examine further whether MDCK‐S, MDCK‐A, and LLC‐MK2D can eliminate contaminating viruses, we passaged clinical specimens containing respiratory viruses other than influenza virus in the three cell lines. The ResPlex II assay did not detect any respiratory virus in the cell‐passaged samples from all cell lines (Table 4). CVEV, RHV, RSVA, RSVB, OC43, and NL63 were eliminated during cell passages irrespective of single or double infection.

**Table 4:** Cell passages of field samples positive for respiratory viruses other than influenza viruses

Group	Target viruses	Clinical samples (positive/total)	MDCK‐S‐passaged samples (positive/total)	MDCK‐A‐passaged samples (positive/total)	LLC‐MK2D‐passaged samples (positive/total)
Double infection 1	ENTV	100% (2/2)	0% (0/2)	0% (0/2)	0% (0/2)
	RHV	100% (2/2)	0% (0/2)	0% (0/2)	0% (0/2)
Double infection 2	ENTV	100% (1/1)	0% (0/1)	0% (0/1)	0% (0/1)
	RSVB	100% (1/1)	0% (0/1)	0% (0/1)	0% (0/1)
Single infection 1	ENTV	100% (3/3)	0% (0/3)	0% (0/3)	0% (0/3)
Single infection 2	OC43	100% (2/2)	0% (0/2)	0% (0/2)	0% (0/2)
Single infection 3	RSVA	100% (2/2)	0% (0/2)	0% (0/2)	0% (0/2)
Single infection 4	NL63	100% (1/1)	0% (0/1)	0% (0/1)	0% (0/1)

## 4 DISCUSSION

In this study, using the ResPlex II assay, we analyzed the specific pattern of co‐infection of respiratory viruses in clinical specimens and evaluated the suitability of three cell lines as a substrate to multiply influenza viruses selectively.

Brunstein et al^17^ have reported some statistically relevant associations between particular pathogens such as CVEV‐RHV (positive correlation) and INFB‐CVEV (negative correlation). These results were reproduced in the present study. Moreover, we newly identified INFA‐INFB as a negatively associated pair and CVEV‐RSVB as a positively associated pair.

We anticipated that in the case of co‐infection, a negative correlation would be observed frequently because the first viral infection would activate interferon signaling and interfere with the second viral infection. However, our study detected significant co‐suppression only in INFA‐INFB and INFB‐CVEV. Influenza viruses did not exhibit remarkable association with most of the other respiratory viruses. One possible explanation for this could be the attenuation of interferon signaling by virally encoded proteins such as influenza virus NS1. It is also possible that the required sample size was larger than that in the present study for detection of more significant associations between influenza viruses and other viruses. These results suggest that co‐existence of influenza virus with other viruses in clinical specimens should not be ignored, and it would thus be necessary to select cell substrates that can eliminate the contaminating viruses and amplify influenza viruses.

Roth et al reported that MDCK 33016PF cells (identical to MDCK‐S cells) could remove adventitious viruses and propagate influenza viruses.^13^ In the present study, we analyzed the property of MDCK‐A and LLC‐MK2D as well as that of MDCK‐S.

The two MDCK cell lines showed different growth phenotypes; MDCK‐S is a suspension cell line, whereas MDCK‐A is an adherent cell line. This difference suggests that MDCK‐S and MDCK‐A have dissimilar patterns of glycosylation and protein expression on the cell surface. Consequently, we expected that MDCK‐S and MDCK‐A would exhibit different profiles of virus amplification; however, MDCK‐S and MDCK‐A showed the same results as both cell lines could propagate all influenza viruses and remove all adventitious agents from multiple infection samples. These results demonstrate that both MDCK‐S and MDCK‐A could be good cell substrates for the preparation of vaccine seed viruses.

Regarding LLC‐MK2D, the isolation rate of influenza viruses was 9.1% (n = 22), whereas that in MDCK‐S and MDCK‐A was 100% with a statistically significant difference (*P* < .01). From four samples in the group of double infection 2, all contaminating CVEV was eradicated during passages in MDCK‐S and MDCK‐A, whereas one CVEV was isolated during LLC‐MK2D passages. These results indicate that LLC‐MK2D might be inferior to MDCK‐S and MDCK‐A considering its low ability to multiply influenza viruses and to eliminate the contaminating viruses.

One of the limitations of this study is that the sample size of the multiple infection groups was small. Larger sample sizes will make it possible to identify more correlations among viruses and to test broader patterns of multiple infections for the selective isolation of influenza viruses. Another limitation is the lack of follow‐up studies to determine whether some viable viruses could be amplified upon additional passages. Cell culture techniques can sometimes multiply viruses even in the PCR‐negative samples.

With respect to the viruses that were not included in the ResPlex II kit, we could not perform tests such as PCR for their detection, due to limited clinical specimens. It was reported that adherent MDCK cells (MDCK‐A) showed no growth of mumps viruses, measles viruses, rubella viruses, herpes simplex viruses, cytomegaloviruses, and parechoviruses^18^; hence, there seems to be little or no risk of amplification of these viruses during passages in MDCK‐S or MDCK‐A.

For the preparation of vaccine seed viruses, reverse genetics can be used as well as clinical specimens. When a novel respiratory virus emerges that can replicate well in MDCK cells with influenza viruses, it might be difficult to prepare seed viruses from clinical specimens. In this situation, reverse genetics is useful because plasmids with no virus are starting materials to produce influenza viruses.

A(H3N2) viruses particularly have serious problems such as low replication efficiency in embryonated hen eggs and critical antigenic alterations during egg adaptation.^19^, ^20^ Recently, the US‐FDA approved an influenza vaccine with cell‐derived seed virus.^21^ It is thus expected that a completely cell‐based A(H3N2) vaccine will pave the way to overcome the difficulties associated with egg‐based vaccines.

MDCK‐S and MDCK‐A cells were determined to be useful in this study for the preparation of vaccine seed viruses without adventitious agents. However, new influenza viruses might appear that have lower abilities to replicate in MDCK cell lines. In this situation, it would be necessary to establish other cell lines and use them together to facilitate the amplification of variable influenza viruses. The use of MDCK‐S, MDCK‐A, and other cell lines could contribute to global public health through the rapid production of safe and effective cell‐based vaccines.
